# Before and after AlphaFold2: An overview of protein structure prediction

**DOI:** 10.3389/fbinf.2023.1120370

**Published:** 2023-02-28

**Authors:** Letícia M. F. Bertoline, Angélica N. Lima, Jose E. Krieger, Samantha K. Teixeira

**Affiliations:** Laboratory of Genetics and Molecular Cardiology, Heart Institute, University of São Paulo Medical School, São Paulo, Brazil

**Keywords:** protein structure prediction, AlphaFold, template-based modeling, free modeling, protein language model

## Abstract

Three-dimensional protein structure is directly correlated with its function and its determination is critical to understanding biological processes and addressing human health and life science problems in general. Although new protein structures are experimentally obtained over time, there is still a large difference between the number of protein sequences placed in Uniprot and those with resolved tertiary structure. In this context, studies have emerged to predict protein structures by methods based on a template or free modeling. In the last years, different methods have been combined to overcome their individual limitations, until the emergence of AlphaFold2, which demonstrated that predicting protein structure with high accuracy at unprecedented scale is possible. Despite its current impact in the field, AlphaFold2 has limitations. Recently, new methods based on protein language models have promised to revolutionize the protein structural biology allowing the discovery of protein structure and function only from evolutionary patterns present on protein sequence. Even though these methods do not reach AlphaFold2 accuracy, they already covered some of its limitations, being able to predict with high accuracy more than 200 million proteins from metagenomic databases. In this mini-review, we provide an overview of the breakthroughs in protein structure prediction before and after AlphaFold2 emergence.

## 1 Introduction

Protein is the term proposed by the Swedish chemist Jacob Berzelius to compounds containing nitrogen and constituted by a combination of amino acids linked by a bond, called peptide bond (Fruton, 1985; Wisniak, 2000; Lehninger et al., 2005; Voet et al., 2014). They are responsible for housekeeping and specific functions essential to life, such as cell structural support, immune protection, enzymatic catalysis, cell signal transduction to transcription and translation regulation (Pearce and Zhang, 2021). The biological function of a protein depends on its tertiary/quaternary structures that derives ultimately from the folding of a polypeptide sequence(s) considering physical chemistry principles and the lowest free energy level, being the understanding of protein folding one of the most important goals in structural biology (Lehninger et al., 2005; Voet et al., 2014).

Experimentally, tertiary protein structures are resolved by X-ray crystallography, nuclear magnetic resonance and electron cryomicroscopy (cryo-EM). Nevertheless, those techniques are complex, time consuming, expensive, and often the structure is not in its native form. Under these constrains, it is no surprise that the number of proteins with resolved tertiary structures is small (200,988 PDB entries) compared to the large number of proteins sequence known (229,580,745 entries on UniProtKB as of January 2023). This is an open challenge waiting for innovative ways to develop novel protein structure prediction approaches such as the ones using computationally models to predict the three dimensional protein structures starting from a polypeptide sequence (Dill et al., 2007; Nassar et al., 2021).

Several algorithms and web servers have been developed with the aim to improve the protein structure prediction. The relevance of these efforts are underscored by the different application that shall be affected including rational drug design, mutational studies and structural comparison, evolutionary analysis, folding studies. Here, we provide an overview of the methods developed to perform protein structure prediction to compare with AlphaFold2, which combines neural networks and homology modeling to generate models that may have experimental accuracy in a growing number of examples. We also discuss its applicability, limitations and efforts to improve the overall algorithm’s performance.

## 2 Structure prediction methods

The prediction methods are usually divided into template-based modeling (TBM) and free modeling (FM), considering the use or not of templates (Gromiha et al., 2018; Bongirwar and Mokhade, 2022; Paiva et al., 2022), even though, recently, some TBM methods use energy-guided model refinement, and part of FM uses fragment-based sampling approaches, extracting information from Protein Data Bank (PDB) through machine learning. Within these two groups, the algorithms developed are usually classified into three different groups: *ab initio* (a FM methodology), threading/fold recognition (a TBM methodology), and homology (a TBM methodology) (Gromiha et al., 2018; Agnihotry et al., 2022). Despite controversial, this classification corresponds to the categories from the Critical Assessment of Structure Prediction (CASP), a biennial competition with the aim to establish the current state of the art in protein structure prediction, which contains three categories: TBM, FM, and an intermediate category, FM/TBM. In this contest, participants submit their models for proteins whose experimental structure has not been published yet (Kryshtafovych et al., 2019).

The *ab initio* approaches are based on the thermodynamics hypothesis that the native protein structure presents the lowest free energy possible (Hardin et al., 2002; Yuan et al., 2003; Gromiha et al., 2018; Agnihotry et al., 2022). The idea of these methods are to predict new folds considering physicochemical properties from the protein fold process, such as hydrogen bonding, contact potential energy, PDB-derived secondary structure propensities, and folding involving both bonded and non-bonded interactions. The *ab initio* method may or may not take into account motif identification in databases using small fragments. Some reviews even divide this category into two distinct methodologies, one dependent and another independent on database information. In this mini-review, we will use the term *ab initio* for methods that model protein structure without a template and that use the laws of thermodynamics as a basis, even though FM methods commonly exploit the information from known structures.

The main advantage of *ab initio* methods is the capacity to obtain novel and unknown protein folds (Dorn et al., 2014). Nevertheless, the complexity of the problem and the high number of conformational possibilities is computationally demanding, limiting the use for long protein sequences. Methods that use fragments may help to reduce the conformational space, but again avoid the prediction of new protein folds. One example of *ab initio* method that considers fragments is QUARK, a template-free novel program developed by Xu and Zhang (2012) in 2012. Briefly, this program breaks the protein sequence into fragments of 20 amino acids, searches for structure in a database, and then, using replica-exchange Monte Carlo simulations, unites the fragments taking into account the force field and energy terms, constituting a complete model.

The threading/fold recognition methodologies are based on the idea that structure is more conserved than the amino acid sequence and has a limited number of protein structure folds in nature (Rost et al., 1997; Dorn et al., 2014; Gromiha et al., 2018; Agnihotry et al., 2022). They consist in choosing the best 3D template of known foldings that fit well in the target sequence considering a scoring function built on pairwise potential, second structure comparison, as well as solvent properties. Thus, the target sequence is aligned with the structure model with the optimal scoring function, reorganizing the atoms of the target sequence in the aligned backbone. Finally, the affinity of the sequence with three-dimensional fold is verified followed by a manual verification. GenTHREADER is a program that uses threading techniques to evaluate the alignments, made using a sequence profile method, and then generates models that will be evaluated by a neural network to give a confidence measure (Jones, 1999).

The homology models derive from the fact that two amino acid sequences that are highly similar have similar structures (Dorn et al., 2014; Gromiha et al., 2018; Agnihotry et al., 2022; Sanjeevi et al., 2022). For this, the target sequence is aligned to a sequence in which the structure is known and an atomic model for the target protein is generated taking into account its similarities with the template backbone, followed by modeling loop regions and sidechains. The final step is to submit the model to energy minimization and evaluate it using the Ramachandran plot. Usually, homology methods achieve protein structures with higher accuracy than other methodologies. But, as other TBM methods, they are limited due to their inability to predict structures for new proteins, as they are dependent on templates. SWISS-MODEL is an automated system that uses homology modeling to predict a three dimensional structure of a protein (Guex et al., 2009; Kiefer et al., 2009). It was the first web server available to generate a 3D protein structure. This program integrates and automates all the processes involved in a homology modeling method, creating a fully automated workflow using a PERL based framework (Kiefer et al., 2009). The program presents an interface friendly for non-bioinformatician users and information, such as PFAM domain annotation and other tools from SWISS-MODEL (Kiefer et al., 2009).

**FIGURE 1:**
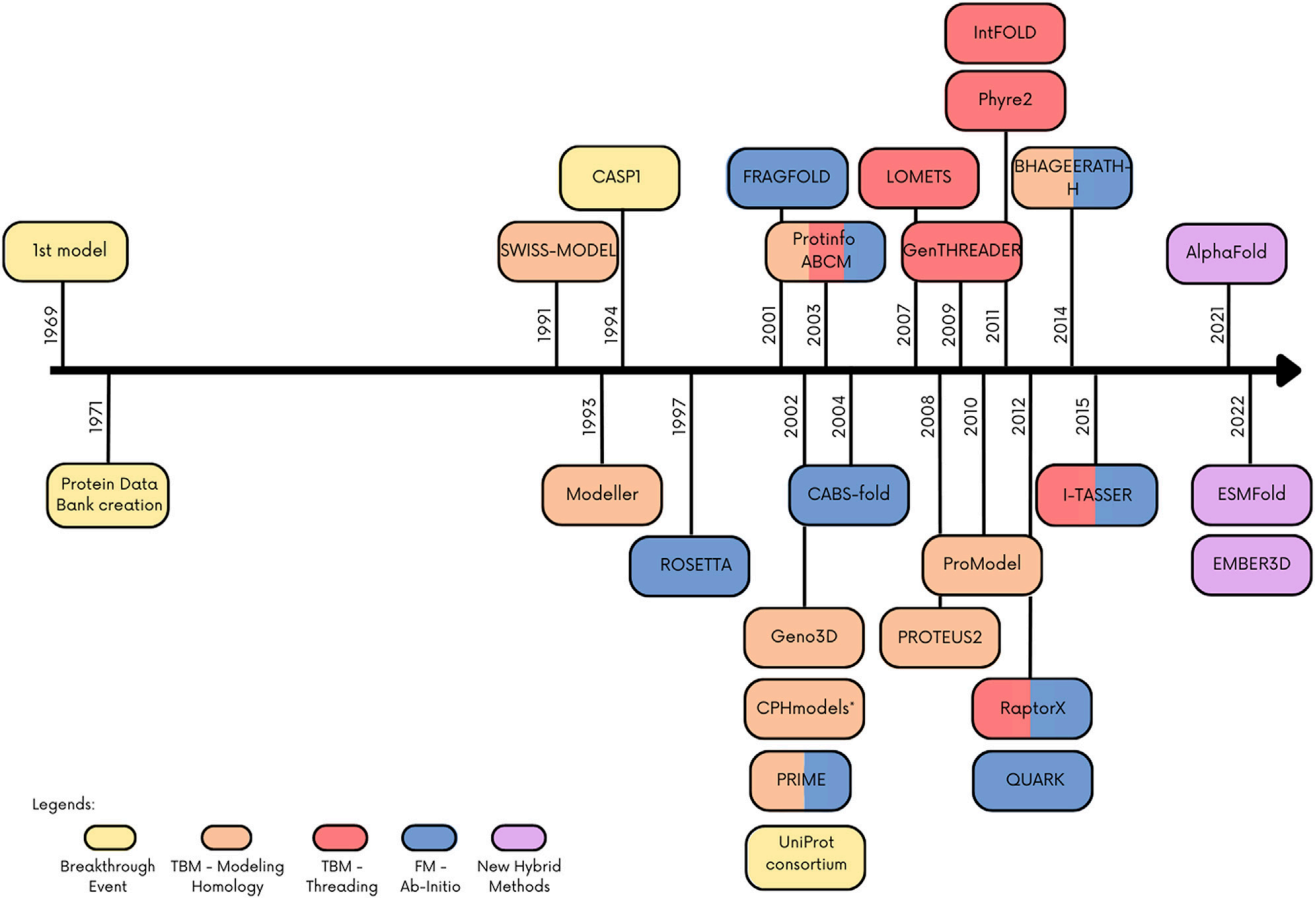
Timeline with main events and programs/webserver in the protein structure prediction. Colored boxes indicate the method or important event in the field.

Recently, new hybrid techniques have been published combining tools or improving known methods with artificial intelligence approaches. This was possible in part due to the development and improvement of computer processing. One of the breakthrough methods that not only combines methodology but also uses artificial intelligence is AlphaFold, an algorithm that beats the other tools in CASP13 and currently represents the state of art in protein structure prediction. Figure 1 depicts a timeline of the emergence of protein structure prediction programs/web servers and their classification considering the groups of methods adopted in this review, as well as other important dates for this field. More comprehensive and detailed reviews about protein structure prediction methods/tools can be found in Paiva et al. (2022), Bongirwar and Mokhade (2022).

## 3 AlphaFold

In 2018, DeepMind, a startup of Google, presented a new software that best performed in the 13th edition of CASP, named AlphaFold. In this competition, AlphaFold achieved the best position in the FM (best-of-five), reaching a summed z-score of 52.8 *versus* 36.6 from the second place and, combining FM and FM/TBM categories, achieved 68.3 z-score *versus* 48.2 (Senior et al., 2019). Even without using a template, AlphaFold also scored well in the TBM category (Senior et al., 2019).

The first version of AlphaFold used deep learning to predict the protein structure, demonstrating that it is possible to learn protein specific potential by training a neural network giving only the protein sequence. It contains a convolutional neural network that is trained by PDB structures to predict the distances between residues, creating distograms. From the amino acid sequence of the target protein, the neural network predicts a distogram based on multiple sequence alignment (MSA) features, in which a separate output from prediction network predicts the probability of backbone torsion distribution. The combined potential obtained by both ends is then optimized by gradient descent, predicting the protein structure itself (Senior et al., 2020).

**FIGURE 2:**
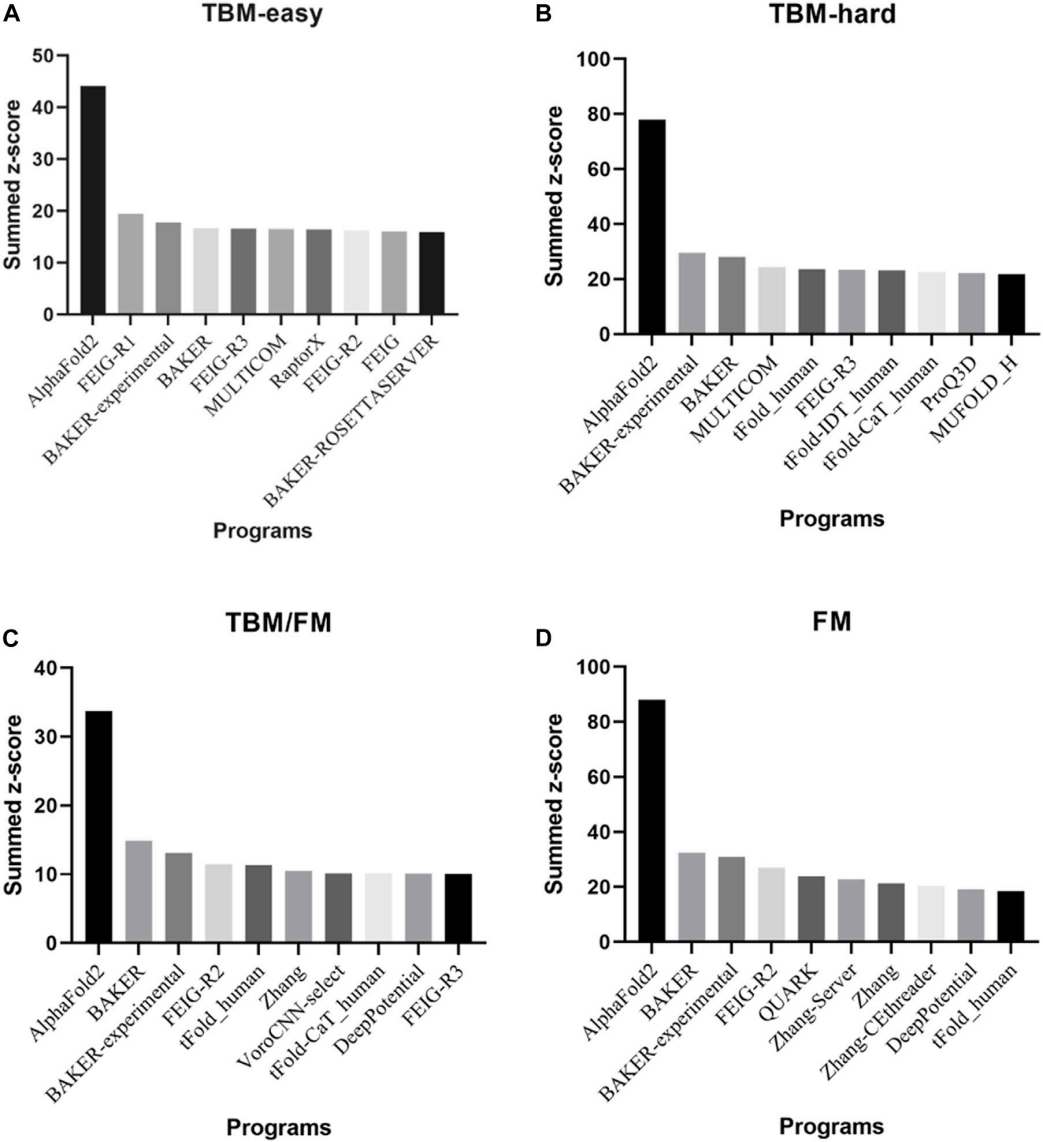
The top 10 programs and/or web servers in CASP14 in **(A)** TBM-easy, **(B)** TBM-hard, **(C)** TBM/FM, and **(D)** FM categories considering summed z-score. Data extracted by CASP official website.

Presented at CASP14 between May and July 2020, AlphaFold2 predicted protein structures with more accuracy than other competing methods, demonstrating a root-mean-square deviation (RMSD) among prediction and experimental backbone structures of 0.8Å *versus* the 2.8Å from the next best performing method. Moreover, AlphaFold2 scored 244.0 in summed z-scores compared with 90.8 for the next closest group (Jumper et al., 2021a; Jumper et al., 2021b). The great performance of AlphaFold2 in all Casp14 categories is depicted in Figure 2.

It is important to emphasize that AlphaFold2 contrasts considerably from the first version of AlphaFold. The authors attribute the high performance of AlphaFold2 by “incorporating novel neural networks architectures and training procedures based on the evolutionary, physical and geometric constraints of protein structures” (Jumper et al., 2021b; Bouatta et al., 2021; Callaway, 2022). AlphaFold2 uses as an input amino acid sequence to construct a MSA based on several databases of protein sequences to determine which parts of the sequence are mutation prone, detecting correlation between them. It also identifies proteins with similar structure with the input that will be used to build an initial representation of the target sequence (template), named as pair representation. Both strategies are not new and are shared by other algorithms in CASP14. Nevertheless, the breakthrough of AlphaFold2 is due to its neural network architectures, more specifically, the two neural network modules, evoformer and the structure module (Jumper et al., 2021b; Oxford Protein Informatics Group, 2021; Skolnick et al., 2021).

The evoformer extracts information from MSA and templates, exchanging information between them in flows back and forth throughout the network, improving the assessment of the MSA, that in turns modifies the protein structures hypothesized by the templates, allowing the MSA and templates in the correct “embedding space”. It consists of two transformers, networks that use attention to boost the speed with which a model can be trained, each of them specialized for a type of data, MSA or pair representations, with a clear communication channel between them. This allows the MSA transformer attention mechanism to incorporate information from the pair representation, adding a bias term from it, augmenting the attention mechanism and allowing it to pinpoint interacting pairs of residues. The pair representation transformer also works in a similar way, but includes an attention to terms of triangles of residues. The structure module, that also contains an attention architecture, uses both representations to prioritize the orientation of the protein backbone, considering the residue rotations and translations, localizing each side chain of each residue in highly constrained within a frame, followed by local refinement and minimization by gradient descent (Jumper et al., 2021b; Oxford Protein Informatics Group, 2021; Skolnick et al., 2021).

Protein-protein interactions are the basis of the biological process, and high-resolution structural characterization of these interactions give rise to insights of their molecular mechanisms and function, as well as direct the design of new drugs that are able to modulate these molecular pathways. Alphafold2 has been used to predict protein-protein interaction, using flexible linkers or artificial gaps and, in general, it predicted heterodimeric protein complexes accurately, exceeding docking approaches usually used in these analysis (Bryant et al., 2022; Yin et al., 2022). Nevertheless, it was limited to predict complexes, such as antigen-antibody and AlphaFold2 accuracy was also limited to predict complexes with protein from different species (Yin et al., 2022). In October 2021, DeepMind extended Alphafold2 to multiple chains - called AlphaFold-Multimer (Evans et al., 2022). For this, AlphaFold-Multimer was trained with protein complexes, and a series of changes in the code were made. The developers observed that performance was better in homomeric than heteromeric interfaces (Jumper et al., 2021b) and it did not predict binding of antigen to antibodies (Yin et al., 2022). Both, AlphaFold2 and AlphaFold-Multimer, are open-source codes and are available on github (https://github.com/deepmind/alphafold).

In the same year, in a partnership between DeepMind and the EMBL-European Bioinformatics Institute (EMBL-EBI), the AlphaFold Protein Structure Database (AlphaFold DB—Available in https://alphafold.ebi.ac.uk) was created, making available over 360,000 predicted structures from 21 organism proteomes (Varadi et al., 2022). Today, AlphaFold DB has over 200 million entries from the human and 47 other organism proteomes, with the structure predictions and their respective analyses freely available to the scientific community. Porta-Pardo et al. (2022) demonstrated that AlphaFold2 increases the structural coverage from 48% to 76% of all human protein residues, dropping the number of human protein without structural coverage from 5027 to 29. Moreover, they quantified that, among the 5027 of the proteins without structure previously, 4459 had structure prediction for over 50% of the protein’s length (88,7%), in which 1408 with high-accuracy (28%). Despite the large amount of data, protein sequences containing non-standard amino acids, like selenocysteine, have been excluded, as well as multiple isoforms codifying by the same gene (Varadi et al., 2022). The database usability is easy, as input, protein name, gene, Uniprot accession number or organism can be used. As output, the AlphaFold DB provides the atomic coordinates in PDB and mmCIF formats and Predicted Aligned Error (PAEs) in JSON format. It is also possible to give feedback about the prediction structure through “Looks great” or “Could be improved” buttons.

Considered as the ground-breaking application of AI in science, AlphaFold has promised to revolutionize structure biology. Its application has been considered to design better protein expression experiment; To solve experimental structures faster, overcoming tedious model building, especially for X-ray crystallography, and to facilitate the interpretation of low-resolution cryo-EM; To protein design and drug development; To examine the effect of mutation in protein function, elucidating their potential impact on human diseases; To provide novel insights in poorly known molecular mechanisms (Perrakis and Sixma, 2021; Porta-Pardo et al., 2022). In this context, Noone et al. (2022) has demonstrated that indeed AlphaFold offers shortcuts to solve protein structures experimentally, predicting the remaining N-terminal region of PTX3 complex, which was, then, validated with cryo-EM class averaging. The hybrid cryoEM/AlphaFold structure allowed the mapping of the putative sites and regions of interaction, giving insights of the functions of PTX3.

However, despite its breakthrough accuracy and performance to predict protein structures, AlphaFold2 models have important limitations. First, AlphaFold2 has difficulty to predict intrinsically disordered proteins/regions (Ruff and Pappu, 2021) and loops (Stevens and He, 2022), especially considering the importance of the latter for drug screening and design, since they are exposed in protein surface and readily available to solvent and other proteins. Ruff and Pappu (2021) demonstrated that residues and regions predicted with low accuracy by AlphaFold2 overlaps intrinsically disordered regions, while Stevens and He (2022) showed that only short loops (<20 amino acids) are predicted with high accuracy by AlphaFold and it has the tendency to over-predict secondary structures in loop regions, usually alpha helix. Both regions are known to be hypervariable and flexible across orthologies, making it difficult to uncover evolutionary constraints from MSA.

Second, AlphaFold2 predicts only a single conformer, not identifying the apo and holo forms. In 67% of a dataset tested, AlphaFold prediction resembled holo form and the proteins were less predictable when the conformational differences between apo and holo forms increased (Saldaño et al., 2022). Moreover, Azzaz et al. (2022) demonstrated that structure prediction of membrane proteins by AlphaFold is not reliable, mainly because it presents inconsistencies in the location of the transmembrane domains. They stress that the protein environment influences the amino acid sequence, imposing folding constraints. These evidences together with the AlphaFold’s inability to predict structures with metal ions, cofactors and other ligands, complexes with DNA or RNA, or post-translational modifications, such as glycosylation, methylation and phosphorylation (Perrakis and Sixma, 2021) highlight the steps to be overcome to improve AphaFold models in drug screening and design. Indeed, Scardino et al. (2023) demonstrated that AlphaFold models showed worse performance in high-throughput docking when compared to their corresponding experimental PDB structures, while Wong et al. (2022) showed that AlphaFold2 protein structure prediction exhibits weak performance on reverse docking in a search for binding targets of bacterial compounds, emphasizing that, even though AlphaFold2 provides rich structural information, more accurate models of protein-ligand interactions are needed to improve use of AlphaFold2 for drug discovery.

Third, AlphaFold fails to predict defects in protein folding due to point mutations. As demonstrated by Buel and Walters (2022), the differences between mutated and wild-type models predicted by AlphaFold are very small, represented by backbones RMSD lower than 1Å. Moreover, Pak et al. (2021) also demonstrated that there is no correlation between AlphaFold accuracy metrics (pLDDT) and the impact of mutations on protein stability change (ΔΔG), neither with the side chain size change.

Finally, of AlphaFold2 cannot predict novel structures, since its algorithm is based on MSA and requires known structures databases. Another important aspect is that the use of evolutionary information from larger MSAs, requiring environmental systems and storage to detect the homology between known and target sequences, demands a powerful computing processors and its structure prediction is time consuming as protein length increases. To overcome this limitation Google offers the Google Colaboratory, which enables access to powerful GPUs. One of these solutions is ColabFold, a fast and easy-to-use software that replaces the AlphaFold2’s homology by MMseq2 (Mirdita et al., 2022), making the computational demands less relevant.

## 4 New methods of protein structure prediction using protein language model

Recently, new free modeling methods have been published to overcome some of the limitations of AlphaFold2, such as the inability to predict novel structures and the necessity of high time and computing processes. These methods are based on protein language models, derived from natural language processing (NLP), which uses the amino acids sequence only and is able to learn evolutionary, structural and functional patterns derived from sequences available in databases, predicting a structural conformation. The idea behind those methods is that the amino acids correspond to words/tokens and proteins to sentences in NLP, assuming that similar semantics come from amino acids that occur in similar contexts.

There are three approaches used in language models: autoregressive, bidirectional, and masked. The first takes into count the previous tokens (amino acids) to predict the probability of a token, the second considers the previous and following tokens independently to estimate the probability of a token, and the last model considers all tokens in a sequence and replaces each token with a mask token. A synthesis of the recent advances in protein language modeling and their applications to protein prediction problems can be found elsewhere (Bepler and Berger, 2021).

Two methods have gained attention this year. ESMfold, developed by Lin et al. (2022) uses a masked transformer protein language model trained in deep information about biological properties, using 15 billion parameters. Compared with AlphaFold2, it did not present the same performance, achieving lower TM-scores (0.68 *versus* 0.85 using AlphaFold2 on CASP14). However, when evaluating AlphaFold2 without MSA, using only the amino acid sequence, ESMFold performed better (0.68 *versus* 0.37 using AlphaFold2 on Casp14). Moreover, it presented an accuracy comparable to AlphaFold2 for structures predicted with high confidence, demonstrating a median all-atom RMSD of 1.91Å and a backbone RMSD of 1.33Å, reaching similar experimental-level accuracy. Finally, this approach also demonstrated a significant improvement in prediction speed, since it does not require the construction of MSA. Using this approach, the authors presented the ESM Metagenomic Atlas, where they predicted more than 617 million structures from metagenomic databases, in which 225 million structures were predicted with high confidence, including those that are novel (Lin et al., 2022).

EMBER2 is a protein language-model developed by Weissenow et al. (2022a), that uses embeddings to predict inter-residue distance (2D structure) introducing attention heads derived from a pre-trained protein language model instead of MSA. Using EMBER2 in *trRosetta* to predict 3D structures is less accurate than AlphaFold2 in predicting a native structure and presents an inferior TM score (0.5 *versus* 0.79 in ColabFold) (Yang et al., 2020). However, it is faster than ColabFold by about 35 fold, similarly to ESMfold. As the comparison made in this work was not fair, since EMBER2 predicts 2D structures and AlphaFold, 3D structures, the authors developed a new approach that uses EMBER2 model, but now applied in three-dimensional structure prediction, named EMBER3D (Weissenow et al., 2022b). Again, Ember3D did not outperform AlphaFold, but is much faster than it and ESMFold. Whereas AlphaFold2 do not perform efficiently in the study of the impact of single amino acids variants into protein structure, Weissenow et al. (2022b) demonstrated that the differences in predicted distances maps generated by EMBER3D correlated well with native and mutant 3D structures from deep mutational scanning, having a better result than ESMfold. Furthermore, they developed a tool that presents the difference between native and mutant predicted structures by all possible amino acid exchanges in each position of a protein sequence. The similarity between de native amino acid and the mutated one helps the identification of exchanges that may cause a high impact on the protein structure.

These new approaches highlight the powerful capacity of language models to identify evolutionary, structural and functional patterns from massive protein sequence databases to solve protein prediction problem, improving prediction speed and requiring less computational power. It is expected that those approaches will develop and gain accuracy with the inclusion of biological knowledge and multi task learning.

## 5 Conclusion

Three-dimensional protein structure determination is important to elucidate the protein function, being critical to understanding biological processes and addressing human health and life science problems in general. Due to the difficulty of determining protein structures by experimental methods, their predictions have been one of the central problems of the scientific community. The advent of AlphaFold2 and the release of millions of protein structures predicted with high accuracy and available in AlphaFold DB allowed an unprecedented expansion of different research fields in life science, impacting the most biological sciences, followed by biochemistry and cell biology, genetics, medical and health sciences and chemical sciences (Varadi and Velankar, 2022).

Protein structure prediction can be applied from understanding the interaction between pathogen and host, how pathogens survive and reproduce, and why they are resistant to certain drugs used, to the development of new and more efficient drugs, reducing the cost in drug discovery and development, as well as the development of new and improved vaccines (Duran-Frigola et al., 2013; Hazra and Patra, 2021). The development of a new vaccine strategy used against COVID-19 by Pfizer, Moderna and Johnson & Johnson, in which the prediction of the mutations and its potential effects in spike protein domain allowed the generation of an immunogen, highlights the window of opportunity that AlphaFold2 offers in the structure-guided vaccine design (Higgins, 2021).

The recent advances in protein structure prediction have contributed to improve protein folding issues. The emergence of AlphaFold2 has taken the problem of protein structure prediction to another level, reaching similar experimental-level accuracy in some cases. Nevertheless, improvements are needed to overcome the limitation to predict novel structures, intrinsically disordered regions and loops, the ability to predict only a single conformer without ligands and still present inconsistencies in its models, and the inability to predict the impact of missense mutation on protein structure. These limitations, ultimately, are important drawbacks to expand the use of AlphaFold in life science.

Efforts are in progress to improve AlphaFold performance and models. Johansson-Åkhe and Wallner (2022) demonstrated that randomly perturbing the neural network weights, forcing it to sample more conformational spaces can improve AlphaFold Multimer performance. Terwilliger et al. (2022) suggested that inclusion of new experimental information can improve parts of the models, showing that application of experimental density maps used iteratively allows the rebuilding of models that can be used as templates by AlphaFold new prediction. Finally, Hekkelman et al. (2022) enriched the models in the AlphaFold DB through transplantation of small molecules and ions based on homologous protein structures. They presented a new resource, the AlphaFill databank, to overcome the limitation presented by AlphaFold models that do not present ligands and co-factors, to help life scientists test new hypotheses and design target experiments.

Simultaneously, new strategies using protein language models are arising to compete with AlphaFold2 in terms of performance and accuracy and to overcome some of its limitations. These strategies, more specific ESMfold, offers an opportunity to identify new proteins and novel functions, allowing the identification of new species, including microorganisms and viruses that endanger human health, as well as those that offer solution to mitigate environmental problems, such as the degradation of polluting and the development of transgenic microorganisms for more efficient product production. For this, ESM Metagenomic Atlas is an important 3D protein structures resource to be deeply investigated by the scientific community (Lin et al., 2022). Finally, the efforts to better predict how mutations affect protein structure using these new approaches are essential to gain insights in human genetic disease and further improve disease management and prediction. Today, these methods do not outperform AlphaFold2, but with the improvement of deep language models and the enrichment of these models with biological information through multi-task learning, they promise to revolutionize the protein structural biology.
